# Removal of Particulate Matter by a Non-Powered Brush Filter Using Electrostatic Forces

**DOI:** 10.3390/toxics11110891

**Published:** 2023-10-30

**Authors:** Jaeseok Heo, Jooyeon Lee, Minyoung Yoon, Duckshin Park

**Affiliations:** 1 Environment Research Institute, Ajou University, Suwon City 16499, Republic of Korea; jsheo1005@nate.com; 2 Department of Transportation Environmental Research, Korea Railroad Research Institute, Uiwang City 16105, Republic of Korea; jooyeon07@krri.re.kr; 3 Environmental Engineering, Inha University, Incheon City 22212, Republic of Korea; opinia0319@gmail.com

**Keywords:** brush filter, bus station, electrostatic force, non-powered, particulate matter

## Abstract

In urban areas, a major source of harmful particulate matter is generated by vehicles. In particular, bus stops, where people often stay for public transportation, generate high concentrations of particulate matter compared to the general atmosphere. In this study, a non-powered type brush filter that generates electrostatic force without using a separate power source was developed to manage the concentration of particulate matter exposed at bus stops, and the removal performance of particulate matter was evaluated. The dust collection performance of the non-motorized brush filter varied by material, with particle removal efficiencies of 82.1 ± 3.4, 76.1 ± 4.7, and 73.7 ± 4.5% for horse hair, nylon, and stainless steel, respectively. In conditions without the fan running to see the effect of airflow, the particle removal efficiency was relatively low at 58.2 ± 8.4, 53.6 ± 9.2, and 58.0 ± 7.3%. Then, to check the dust collection performance according to the density, the number of brushes was increased to densify the density, and the horse hair, nylon, and stainless steel brush filters showed a maximum dust collection performance of 89.6 ± 2.2, 88.3 ± 3.2, and 82.1 ± 3.8%, respectively. To determine the replacement cycle of the non-powered brush filter, the particulate removal performance was initially 88.0 ± 3.2% when five horse hair brushes were used. Over time, particulate matter tended to gradually decrease, but after a period of time, particulate matter tended to increase again. The purpose of this study is to evaluate the particulate matter removal performance using a brush filter that generates electrostatic force without a separate power source. This study’s brush filter is expected to solve the maintenance problems caused by the purchase and frequent replacement of expensive HEPA filters that occur with existing abatement devices, and the ozone problems caused by abatement devices that use high voltages.

## 1. Introduction

Particulate matter is harmful to human health [1,2,3,4,5,6]. Vehicles are a representative source of particulate matter in urban areas [3,7,8]. Internal combustion engines in vehicles emit various pollutants, including particulate matter, sulfur oxides, nitrates, and ozone [6,7]. The particulate matter generated by internal combustion engines can lead to cardiovascular contraction, causing hypertension and heart disease [6,9,10]. Studies have shown that high, continuous exposure to particulate matter is associated with an increased relative risk of daily cardiovascular disease mortality [6,11,12,13]. Styer et al. [14] revealed the adverse effects of particulate matter concentrations on human health, with every 10 μg/m^3^ increase in PM_10_ concentration increasing mortality by 0.3%, excluding natural deaths. Samet et al. [15] found that an increase in PM_10_ concentrations up to 10 μg/m^3^ increased cardiorespiratory mortality by about 0.7%. Yin et al. [6,16] reported that every 10 μg/m^3^ increase in particulate matter concentration increased death from cardiopulmonary disease by 0.62% (0.43–0.81%). Other studies have noted that PM_2.5_ can penetrate the lungs through inhalation and cause adverse health effects such as local and systemic inflammation [1,17,18,19,20]. In light of these findings, reducing atmospheric PM_10_ levels would reduce harm to human health [6].

According to Korean Statistical Information Service data, in 2020, the number of weekly public transportation users in Seoul is approximately 7.6 million, and the number of uses per person is 9.86, of which 2.81 are city buses only, 3.92 are subway only, and 3.13 are combined [21]. Li et al. [22] and Zheng et al. [23] reported that many people use buses for transportation and wait at bus stops for more than 10 min every day. Moreover, Lee et al. [24] reported higher exposure to particulate matter at bus stops near roads than other locations because buses frequently stop and depart, while pedestrians and bus users remain present for around 10 min when getting on and off the bus. In urban areas, people may be exposed to high pollutant concentrations as they wait to catch a bus [25]. Hess et al. [26] found that people were exposed to particulate matter while waiting in and around bus shelters. Particulate matter concentrations were consistently higher inside bus shelters with road-facing openings than outside the same shelters [8,26]. Thus, people are exposed to exhaust gases and particulate matter generated by vehicles for a long period of time when using buses for transportation [6].

To reduce exposure to particulate matter in bus shelters, previous studies have generated electrostatic charge by rotating brushes. To achieve it, a motor is used, and the rotational force of the motor rotates the brush to friction with the PVC plate. In this case, a study was conducted to identify the difference in electrostatic charge generated by different rotation speeds, select the most suitable material and speed, and remove atmospheric particulate matter using electrostatic charge [6]. Based on this research, bus shelters were fitted with brush filters in Bucheon and Guri city to study the reduction in particulate matter entering the bus shelters [27].

When outdoor structures such as bus shelters are retrofitted with a brush filter, a power source must be supplied to drive the motor, and relevant electrical designs and subsequent construction are needed. This additional construction carries a cost, which limits the installation of particulate matter reduction systems using brush filters at existing bus shelters. 

To solve this problem, a brush filter that generates electrostatic forces from only airflow (without a motor) was studied to enable the installation of filters without additional electrical work. Depending on airflow, the brushes in the module rub against friction rods to generate electrostatic force. To evaluate particulate removal performance based on brush material, several brush filters made of horse hair, nylon, and stainless steel were tested. As in the previous study, the non-powered brush filter was modularized to enable easy maintenance, such as replacement and cleaning of the brush filter. This study was conducted at the laboratory scale with airflow generated using a fan, and the particulate matter removal performance of the brush filter was confirmed.

## 2. Materials and Methods

### 2.1. Measurement of Electrostatic Force According to Brush Material

To generate electrostatic force, the triboelectrification principle was used. Polyvinyl chloride (PVC) was selected for the friction plate material based on the results of previous research [6], and horse hair, nylon, and stainless steel were selected as brush filter materials based on the order of electrification reported in the literature (Table 1) [28,29,30,31,32].

In the experimental device, a thin friction rod made contact with the brush and a large area was provided to increase friction with the brush at the bottom of the module. In total, 95 thin friction rods with a diameter of 5 mm were installed at the bottom of the module and a brush was installed at the top to measure the electrostatic force. The electrostatic force generated was measured using an electrostatic sensor (ARS-S005, Dong Il Technology Ltd., Hwaseong City, Republic of Korea). During measurement, an electrostatic sensor was installed at a distance of approximately 50 mm from the brush filter. The electrostatic forces generated with the horse hair, nylon, and stainless steel brush filters were measured. To compare the electrostatic force under difference airflows, the change in electrostatic force generated when the fan was set to the first, second, and third settings was measured.

### 2.2. Removal Performance of Particulate Matter according to Brush Filter Material 

Next, the effect of the electrostatic forces measured in the first experiment on the filter’s performance in removing particulates was determined. The particulate matter removal performances of horse hair, nylon, and stainless steel brush filters were measured while the fan was turned on (to generate electrostatic forces) or off. To replicate particulate matter, A1 Ultrafine Test Dust (A1 dust, Powder Technology Inc., Arden Hills, MN, USA) was used. The dust was sprayed into the duct using a solid aerosol generator (SAG 410, Topas GmbH, Dresden, Germany). The duct used in the experiment was 10 × 75 × 200 cm. The distance between the filter and the location of spraying particulate matter was 100 cm, and the inlet was measured at a distance of 75 cm from the location of spraying particulate matter to ensure that the sprayed particulate matter was measured as evenly as possible. The concentration was measured with a portable aerosol spectrometer (1.109 (ver. 11-D), Durag Group, Hamburg, Germany). During the experiment, the concentration of A1 Ultrafine Test Dust in the duct was maintained at an average of 300 μg/m^3^. Measurement equipment was installed at the front and rear of the brush to measure the particulate matter removal performance of the brush filter. The particulate matter reduction efficiency (%) was calculated using Equation (1).

(1) $$U=\left(1-\frac{C_{out}}{C_{in}}\right)\times 100$$

Here, the particulate matter reduction efficiency is *U*, the particulate matter concentration at the front of the brush filter is *C_in_*, and the particulate matter concentration at the rear is *C_out_*. The experiment confirmed the particulate matter collection performance attributable to the electrostatic forces generated with PVC as the friction rod material and horse hair, nylon, and stainless steel as brush filter materials.

### 2.3. Removal Performance of Particulate Matter according to Brush Filter Density

**Figure 1:**
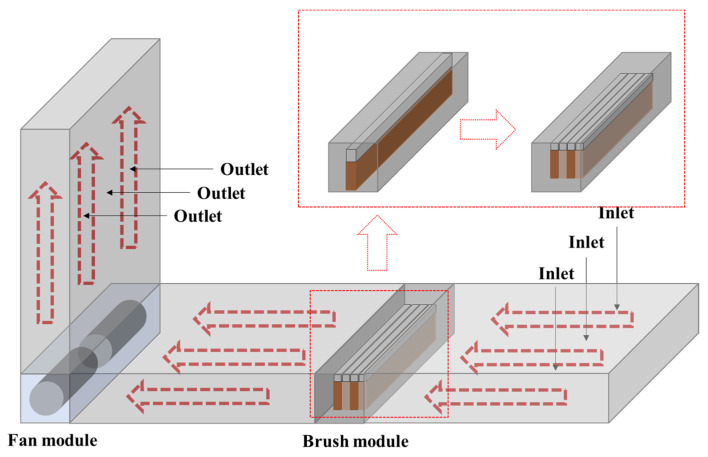
Experiment apparatus diagram for brush filter density variation.

To improve the particulate matter collection performance of the brush filter, brush filters made of horse hair, nylon, and stainless steel were installed in groups of one to five to compare their particulate matter collection performance. Figure 1 shows the experimental setup used to test the brush filters of each material and brush filter density.

### 2.4. Calculation of the Brush Filter Replacement Cycle 

High-efficiency particulate air (HEPA) filters, which are commonly used in commercial products, are effective at capturing particulate matter but carry high maintenance costs due to their high price and short replacement cycle [33]. Selecting a filter suitable for a given use based on the reduction device cost, filter replacement cost, and replacement cycle is essential.

**Figure 2:**
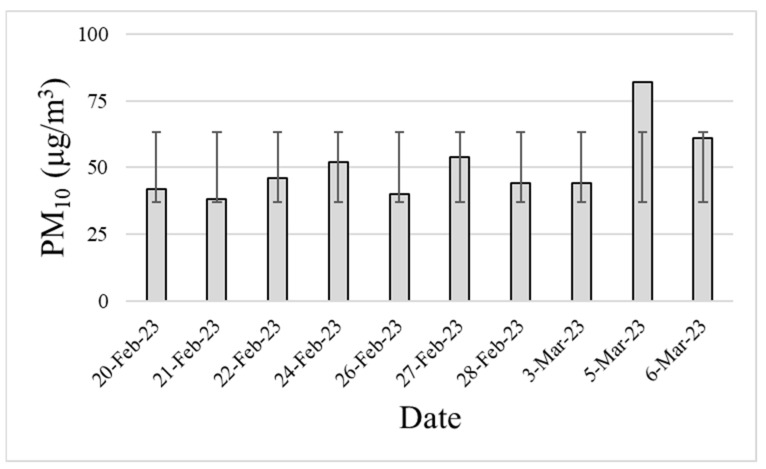
Atmospheric PM_10_ concentrations measured at Bugok 3-dong measurement station during the experimental period in 2023.

In this study, to calculate the replacement cycle for the non-powered brush filter proposed as an alternative to HEPA filters, an experiment was conducted under adverse conditions in terms of the particulate matter concentration in the air. To set these conditions, the ambient particulate matter concentrations were obtained from public data collected at the Bugok 3-dong monitoring station (South Korea). Figure 2 shows the PM_10_ concentrations. The experiment to calculate the replacement cycle was conducted by spraying 300–350 μg/m^3^ of A1 Ultrafine Test Dust into the duct, which was six times greater than the average ambient atmospheric particulate matter concentration (50.3 μg/m^3^). The brush filter was measured using a module composed of five brushes made of horse hair, which had yielded the best results in the brush density experiment. The measurement period was 6 h per day for a total of 10 days. During this period, the replacement cycle was determined based on the reduction in efficiency of particulate matter removal over time.

## 3. Results

### 3.1. Electrostatic Force Generation according to Brush Material

**Figure 3:**
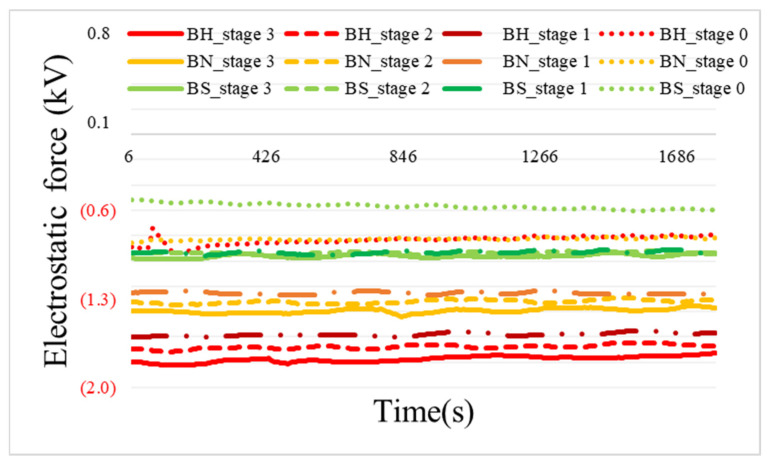
Comparison of the electrostatic force (kV) generated according to brush material (BH, horse hair; BN, nylon; BS, stainless steel) under different air velocity (fan stages 0 = 0, 1 = 0.6, 2 = 0.8, 3 = 1.0 m/s).

Figure 3 shows the results of the electrostatic force experiment at different air velocities for filters made of horse hair, nylon, or stainless steel, using a PVC friction plate.

When the fan was not operated (stage 0), the electrostatic forces generated with horse hair, nylon, and stainless steel brushes were −0.84, −0.83, and −0.57 kV, respectively (Figure 3). The electrostatic force measured in the absence of airflow is due to the residual electrostatic force generated by the previously generated airflow even after the fan was stopped. The highest electrostatic forces of −1.78 ± 0.02 kV for horse hair, −1.39 ± 0.02 kV for nylon, and −0.96 ± 0.01 kV for stainless steel brushes were measured when the fan was set to the highest setting (stage 3). As noted in Table 1, the electrostatic force gradually decreases in the order of wool (similar to horse hair), nylon, and metal (similar to stainless steel), according to the amount of charge inherent in the material. In addition, the electrostatic force generated with each material gradually decreased as the fan setting decreased, but this decrease was not significant. This result confirmed that when air velocity is present, friction between the brush and friction plate generates an electrostatic force proportional to the intrinsic charge of each material. Table 2 shows the electrostatic forces generated with each brush material and air velocity setting in detail.

### 3.2. Particulate Matter Removal Performance according to Brush Filter Material

**Figure 4:**
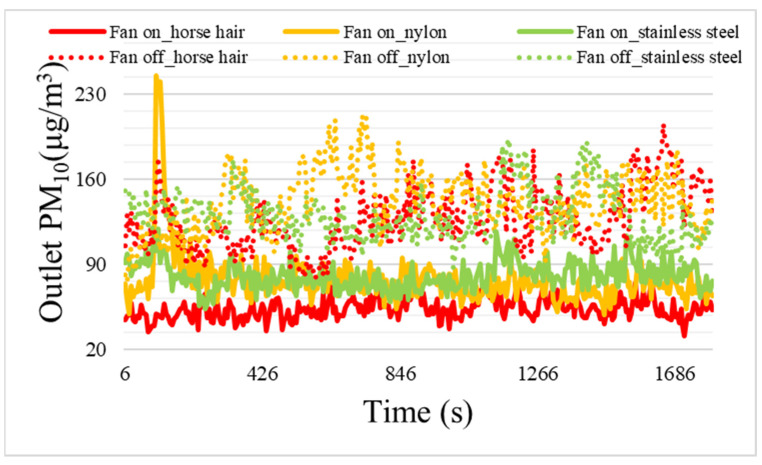
Comparison of the outlet PM_10_ concentrations according to brush material in the presence (On) or absence (Off) of air velocity.

To assess the particulate matter collection performance of the brush filters, A1 Ultrafine Test Dust was sprayed into the duct at a concentration of approximately 300 μg/m^3^. For this experiment, one brush was installed in the module. Figure 4 shows the particulate matter concentrations according to brush material and the presence or absence of airflow. To check the structural effect of the brush filter on particulate matter reduction performance, the efficiency of horse hair, nylon, and stainless steel brushes was 58.2, 53.6, and 58.0%, respectively, when the fan was turned off. When fans were operated to generate electrostatic force, the horse hair, nylon, and stainless steel brushes had efficiencies of 82.1, 76.1, and 73.7%, respectively. These results confirmed that electrostatic force generation improved atmospheric particulate matter removal, via collection on the brush filter, regardless of brush material. Table 3 shows the mean and standard deviation of the particulate matter concentration in the duct and the brush efficiency and standard deviation according to each material and the presence (On) or absence (Off) of airflow. These results are similar to the outcomes of the electrostatic force measurements. Overall, particulate matter collection performance varied according to the magnitude of the electrostatic force generated by each brush. Thus, electrostatic force generation impacts particulate matter collection.

To determine whether electrostatic force has a significant effect on particulate matter reduction, the particulate matter reduction efficiency by particle diameter was analyzed and compared among filters. The direct source of the reduction was inferred based on the differences in the removal efficiencies by particle diameter between measurements before and after electrostatic force was generated.

**Figure 5:**
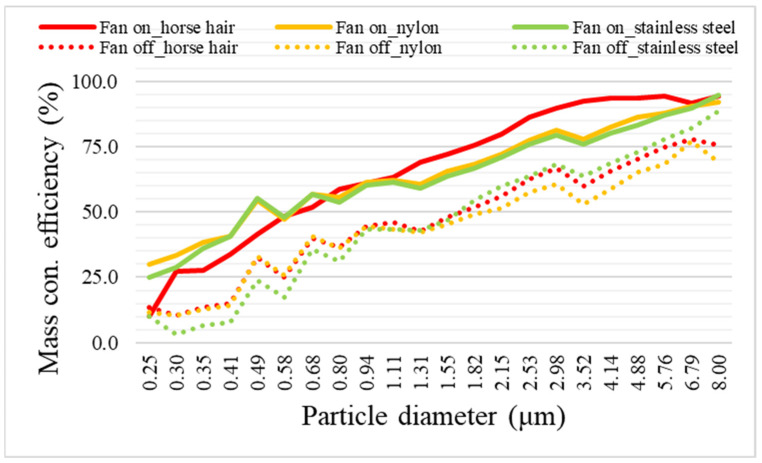
Comparison of the particulate matter removal efficiency by particle size according to the brush material in the presence (On) or absence (Off) of air velocity.

Studies have shown that the generation of an electrostatic force on a filter can increase its filtration efficiency without increasing the filter pressure drop [6,34,35]. Similarly, in this study, the fine dust removal performance by particle diameter improved when electrostatic force was generated [6]. Figure 5 shows the changes in the particulate matter removal efficiency by particle diameter according to brush material. Even for particles smaller than PM_1_, each brush filter showed at least a doubling in its reduction efficiency when electrostatic force was applied. In particular, for PM_2.5_ and larger particles, the horse hair brush filter had reduction efficiencies of at least 80%. Based on these results, among the brush materials, the horse hair brush filter offered the best particulate matter reduction performance according to particle diameter.

### 3.3. Particulate Matter Removal Performance according to Brush Filter Density

**Figure 6:**
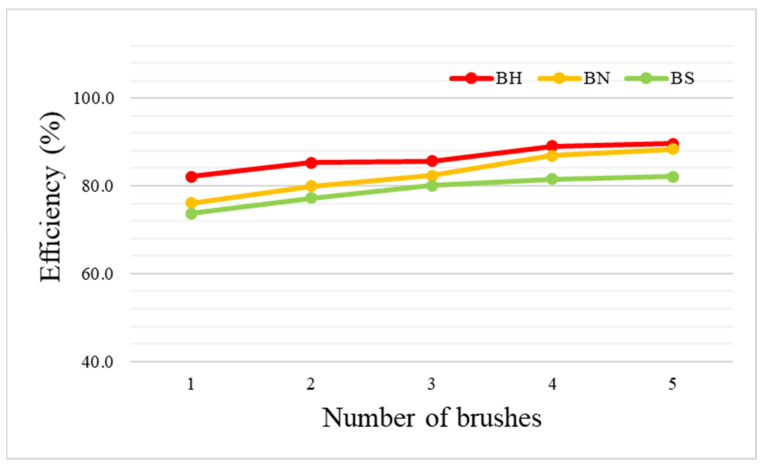
Comparison of the PM removal efficiencies of each type of brush (BH, horse hair; BN, nylon; BS, stainless steel) according to the brush density.

Next, the brush density was varied by varying the number of brushes in the module (Figure 1), and the particle removal performance was evaluated by varying the brush density and material (Figure 6). Table 4 shows the mean and standard deviation of particulate matter concentrations in the duct and the brush efficiency and standard deviation according to each material and density. The efficiency of each brush type improved as the brush density increased; however, the improvements between the lowest (one brush) and highest (five brushes) densities were not large, with differences of about 10%. Overall, the module containing five horse hair brushes had the highest reduction efficiency (89.6 ± 2.2%), which decreased with a reduction in brush density to 89.1 ± 2.3, 85.6 ± 2.5, 85.3 ± 2.7, and 82.1 ± 3.4%, respectively. Nylon brushes, which showed moderate performance, had reduction in efficiencies of 88.3 ± 3.2% for five brushes to 76.1 ± 4.7% for one brush. Finally, stainless steel showed the lowest A1 Ultrafine Test Dust collection performances of 82.1 ± 3.8% for five brushes to 73.7 ± 4.5% for one brush. The results show that the efficiency gradually improves by 2–3% as the brush density increases, but it seems that the efficiency is improved by structurally removing particles due to the density of the brush, rather than improving the dust collection performance by electrostatic forces, since the electrostatic charge generated per unit area in the brush is the same.

### 3.4. Calculation of the Brush Filter Replacement Cycle

**Figure 7:**
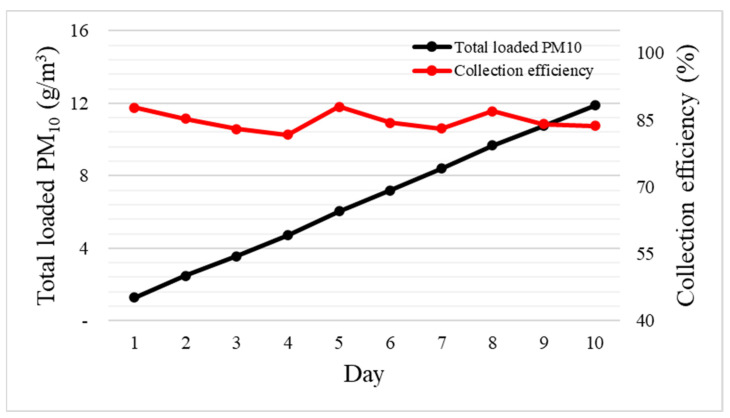
Changes in the horse hair brush filter efficiency and particle accumulation on the filter over time.

To determine the replacement cycle for the brush filter, the brush filter was operated for 10 days under conditions higher than outdoor particulate matter concentrations (300–350 μg/m^3^), and the filter efficiency and total PM_10_ load were measured (Figure 7). On day 1, the filter had a high A1 Ultrafine Test Dust removal efficiency of 88.0 ± 3.2%. Subsequently, the efficiency gradually decreased but then increased as the total PM_10_ load increased. The replacement cycle was determined as the time at which the particle removal performance of the horse hair brush filter fell below 80%. However, the experiment revealed that the A1 Ultrafine Test Dust removal efficiency first decreased (as particles initially accumulated) and then increased (as accumulated particles fell off the filter).

The results presented in Figure 7 and Table 5 indicate that the brush filter can be used semi-permanently provided that the particles accumulated within the duct are periodically removed. Table 6 also shows the amount of A1 Ultrafine Test Dust accumulated in the filter over time. It is simply calculated as the difference between the quantity of A1 Ultrafine Test Dust entering the filter and the quantity of A1 Ultrafine Test Dust exiting the filter.

## 4. Conclusions

In this study, we assessed the efficacy of an outdoor air purification system with different brush materials and densities to reduce exposure to fine dust at bus shelters; this system reduces particulate matter using the electrostatic force generated by the friction of the brush.

Among the brush materials assessed, the horse hair brush generated about −1.8 kV of electrostatic force, which was greater than those generated by nylon and stainless steel. The A1 Ultrafine Test Dust removal efficiencies were 82.1 ± 3.4, 76.1 ± 4.7, and 73.7 ± 4.5% for the horse hair, nylon, and stainless steel brush filters, respectively. Furthermore, the A1 Ultrafine Test Dust removal efficiencies increased with an increasing number of brushes installed, reaching maximum efficiencies of 89.6 ± 2.2, 88.3 ± 3.2, and 82.1 ± 3.8% for five horse hair, nylon, and stainless steel brushes, respectively. Evaluation of the brush replacement cycle indicated that the brush filter can be used semi-permanently. These results confirm the potential utility of an electrostatic particulate matter collection system, and demonstrate that the use of a non-powered brush filter can effectively reduce atmospheric particulate matter concentrations.

The study used a laboratory-scale experimental design that was conducted indoors. As a result, the characteristics of the external dust (size, shape, composition, etc.) and environmental factors (temperature, humidity, wind speed, etc.) could not be considered. Due to these limitations, a design should consider the environment in the field. In particular, it is necessary to consider ambient relative humidity, which is a limiting factor for static electricity generation. Further research is needed on brush filters that can be operated in high ambient relative humidity in the field by controlling the humidity in the module. This can maintain the dry condition of the brushes in the module, so that the brush filter can be used in field conditions of high ambient relative humidity.

## Figures and Tables

**Table 1:** Order of electrification according to materials reported in the literature.

Most Positive → Most Negative	Publication Year and Reference
wool → nylon → cotton → silk → PVC → PE → PTFE	1955
nylon → wool → silk → cotton → NR → S → PE → PVC → PTFE	1962
nylon → wool → silk → paper → cotton → PE → PP → PVC → Si → PTFE	1987
quartz → nylon → wool → silk → cotton → paper → metals → rubber → PTFE → PVC	1998
fur → glass → silk → wood → rubber → plastic	2015
Copy paper → nylon → PP → quartz → PE → PDMS → PTFE → PVC	2019
wool → PP → silk → nylon→ NR → cellulose → Al → Si → quartz → S → PE → PTFE → PDMS → PVC	2022

Abbreviations: PP, polypropylene; NR, polyisoprene (i.e., natural rubber); quartz, c-SiO_2_; PE, polyethylene; PTFE, polytetrafluoroethylene; PDMS, polydimethylsiloxane (i.e., silicone rubber); PVC, polyvinyl chloride.

**Table 2:** Electrostatic force (kV) generated for each brush material and flow velocity.

Friction Plate	Flow Velocity (m/s)	Horse Hair (kV)	Nylon (kV)	Stainless Steel (kV)
PVC	0	−0.84 ± 0.03	−0.83 ± 0.01	−0.57 ± 0.02
	0.6	−1.58 ± 0.01	−1.25 ± 0.01	−0.93 ± 0.01
	0.8	−1.68 ± 0.02	−1.32 ± 0.01	−0.94 ± 0.01
	1.0	−1.78 ± 0.02	−1.39 ± 0.02	−0.96 ± 0.01

**Table 3:** Average particulate matter removal by each brush filter in the presence or absence of air velocity.

		Horse Hair	Nylon	Stainless
Fan on	Inlet (μg/m^3^)	298.2 ± 36.4	323.2 ± 176.1	307.9 ± 53.4
	Outlet (μg/m^3^)	53.3 ± 8.5	77.4 ± 22.3	80.8 ± 11.7
	Efficiency (%)	82.1 ± 3.4	76.1 ± 4.7	73.7 ± 4.5
Fan off	Inlet (μg/m^3^)	304.8 ± 39.9	305.3 ± 47	311.6 ± 36.5
	Outlet (μg/m^3^)	127.3 ± 25.9	141.5 ± 24.8	130.8 ± 21.2
	Efficiency (%)	58.2 ± 8.4	53.6 ± 9.2	58.0 ± 7.3

**Table 4:** Average PM removal by each type of brush filter according to the brush density.

Brush Type	Number of Brushes	Inlet (μg/m^3^)	Outlet (μg/m^3^)	Efficiency (%)
Horse hair	1	298.2 ± 36.4	53.3 ± 8.5	82.1 ± 3.4
	2	319.2 ± 42.8	46.9 ± 7.8	85.3 ± 2.7
	3	321.6 ± 42.6	46.3 ± 7.1	85.6 ± 2.5
	4	299.5 ± 41.6	32.7 ± 5.2	89.1 ± 2.3
	5	325.7 ± 50.6	33.8 ± 4.7	89.6 ± 2.2
Nylon	1	323.2 ± 176.1	77.4 ± 22.3	76.1 ± 4.7
	2	309.5 ± 41.8	62.0 ± 8.9	80.0 ± 4.2
	3	314.6 ± 53.6	55.4 ± 8.5	82.4 ± 3.5
	4	310.3 ± 46.6	40.7 ± 8.2	86.9 ± 3.1
	5	332.9 ± 63.6	38.9 ± 8.0	88.3 ± 3.2
Stainless steel	1	307.9 ± 53.4	80.8 ± 11.7	73.7 ± 4.5
	2	312.3 ± 43.6	71.1 ± 13.4	77.2 ± 5.1
	3	316.7 ± 51.1	63.2 ± 9.6	80.0 ± 3.6
	4	261.3 ± 32.5	48.1 ± 9.9	81.6 ± 4.2
	5	307.6 ± 179.3	55.2 ± 10.7	82.1 ± 3.8

**Table 5:** Changes in the PM_10_ removal efficiency of the horse hair brush filter over time.

Day	1	2	3	4	5	6	7	8	9	10
Efficiency (%)	88 ± 3.2	85 ± 3.2	83 ± 4.7	82 ± 4.0	88 ± 3.3	84 ± 3.9	83 ± 4.0	87 ± 3.2	84 ± 5.1	84 ± 3.9

**Table 6:** Total accumulation of PM_10_ collected by the horse hair brush filter over time.

Day	1	2	3	4	5	6	7	8	9	10
Holding capacity (g/m^3^)	1.27	2.49	3.55	4.73	6.03	7.19	8.39	9.68	10.77	11.89

## Data Availability

Not applicable.
